# Prenatal Caffeine Exposure Impairs Pregnancy in Rats

**DOI:** 10.22074/ijfs.2015.4616

**Published:** 2015-12-23

**Authors:** Maryam Yadegari, Mozafar Khazaei, Morteza Anvari, Mohadeseh Eskandari

**Affiliations:** 1 Department of Biology and Anatomical Sciences, Shahid Sadoughi University of Medical Sciences, Faculty of Medicine, Yazd, Iran; 2 Fertility and Infertility Research Center, Kermanshah University of Medical Sciences, Kermanshah, Iran

**Keywords:** Caffeine, Rat, Implantation

## Abstract

**Background:**

In recent years, concerns have been raised about human reproductive disorders. Caffeine consumption is increasing by the world’s population and there is a relationship between caffeine intake and adverse reproductive outcomes. The aim of this study was to evaluate the effects of caffeine on implantation sites, number of live births, birth weight, crown-rump length (CRL) and abnormality in pregnant rats.

**Materials and Methods:**

In this experimental study, 40 female albino rats (170-190 g) were randomly divided into two experimental and two control groups (n=10/each group). In both experimental groups, animals received caffeine intraperitoneally (IP: 150 mg/kg/day) on days 1-5 of pregnancy. In experimental group 1, treated animals were euthanized on day 7of pregnancy and the number of implantation sites was counted. In experimental group 2, treated animals maintained pregnant and after delivery, the number of live births, birth weight, CRL and abnormality of neonates were investigated. In control group, animals received IP injections of distilled water. Data were analyzed by independent t test.

**Results:**

Results showed that administration of caffeine significantly decreased the number of implantation sites, number of live births and CRL as compared with control group (P<0.05). There were no significant differences regarding birth weight and abnormality of neonate rats between experimental and control groups.

**Conclusion:**

These results suggest that caffeine caused anti-fertility effect and significantly decreased CRL in neonate rats.

## Introduction

Coffee, tea, chocolate, as well as certain medications are the major sources of caffeine (1,3). A variety of physiological adaptations are required to create an environment for the optimal fetal development during pregnancy. Pregnant women are concerned about what they consume during pregnancy. The widespread consumption of caffeine by pregnant women suggests that it is important to determine whether caffeine may influence maternal physiology and development of the fetus during pregnancy (1,2). 

A few of the known biological effects of caffeine are central nervous system stimulation, increased secretion of catecholamine, increased heart rate and relaxation of smooth muscle (4,6). Due to high lipid solubility and low molecular weight characteristics, caffeine crosses through the placenta easily (4). Fenster et al. (3) reported that after repeated consumption of caffeine in pregnant women, plasma caffeine levels and its biological effects are influenced by the metabolic characteristic of caffeine. An increase in half-life of caffeine during the last few weeks of pregnancy is caused by the elevated estrogen levels. Therefore, the caffeine blood levels in the mother and the fetus increase, but the fetus has no enzymes to metabolize it (2,7). A number of studies have showed that caffeine may influence the reproductive outcome and fetal development (1,3). 

Previous studies indicated that maternal caffeine consumption might increase the risk of an early spontaneous abortion (8,9). Other studies showed that consumption of coffee during pregnancy might be associated with a shortened gestation and a lowered birth weight, but they did not find any relationship between risk of spontaneous abortion and caffeine metabolite (8,10). 

Some investigators reported that consuming high amounts of caffeine during pregnancy may be harmful (2,6). It is yet unknown whether consuming small amounts of caffeine during pregnancy affects fetal development. In mammals, caffeine has been shown to be teratogenic only at extremely high doses, or following a single large intraperitoneal (IP) injection. Reduced fetal body weight and delayed skeletal ossification have been observed at relatively high doses of caffeine (2,4,8). 

Since caffeine-containing products are consumed in large quantities, its effect during gestation on the developing offspring is important (11). Although there are studies focusing on the effects of caffeine, the exact activity mechanisms of caffeine on pregnancy have been less characterized (2,4,8). In the present study, the effects of caffeine injection on the offspring of pregnant rats given a dose approximating to that of a high daily coffee consumption were evaluated. Furthermore we aimed to investigate the effects of caffeine on implantation sites, number of live births, birth weight, crown-rump length (CRL) and abnormality of neonate rats. 

## Materials and Methods

### Acute toxic dose

Acute toxicity [lethal dose 50 (LD50)] of caffeine was evaluated through the IP injections into rats as described by Miller and Tainted (12). Briefly, the method involved the administration of 7 different concentrations of caffeine to 7 groups of rats (n=5/group) in pilot study. After 1 week, there were no deaths in animals receiving caffeine in concentrations of 1, 10, 50, 100, 150, 500 and 1000 mg/kg. The effects of 3 different concentrations of caffeine on fertility rate were tested. The 50 and 100 mg/kg/day showed no significant change in fertility rate; therefore, the dose was changed to 150 mg/kg/day. 

### Treatment

This experimental study was carried out between April to June 2012 at the Fertility and Infertility Research Center, Kermanshah University of Medical Sciences, Kermanshah, Iran. The Ethical Committee of Kermanshah University of Medical Sciences approved all procedures used in this study. 

In present study, 40 young female albino rats (170- 190 g) were used. Prior to mating, the females were isolated for one month to rule out pre-existing pregnancy. Female rat were mated with males (3:1) in each cage, under controlled environmental conditions with a 12/12 hour light-dark cycle and free access to food. In the next morning, a positive sign of mating was confirmed by sperm-positive vaginal smears and the presence of copulatory plugs (13). The day on which a vaginal plug was found was designated as day 0 of gestation. Then, the positive vaginal smear rats were classified into two experimental (groups 1 and 2) and two control groups. Both experimental groups (n=10/each) received the IP injections of caffeine in concentration of 150 mg/kg/day on days 1-5 of pregnancy, and both control groups (n=10/each) received the IP injections of distilled water. In experimental group 1, animals were maintained on normal diet and on day 7 of pregnancy, they were euthanized. 

The number of implantation sites in each uterine horn was determined under a stereomicroscope (Leica, Germany) and compared with the control group. In experiment group 2, animals were maintained pregnant till delivery. After delivery, the number of live births, birth weight and CRL of neonates were investigated. Abnormality of neonates was investigated under a stereomicroscope and compared with the control group. 

### Statistical analysis

The data were analyzed by independent–t test using the SPSS (SPSS Inc., USA) version 15. A value of P<0.05 was considered significant. 

## Results

**Fig.1:**
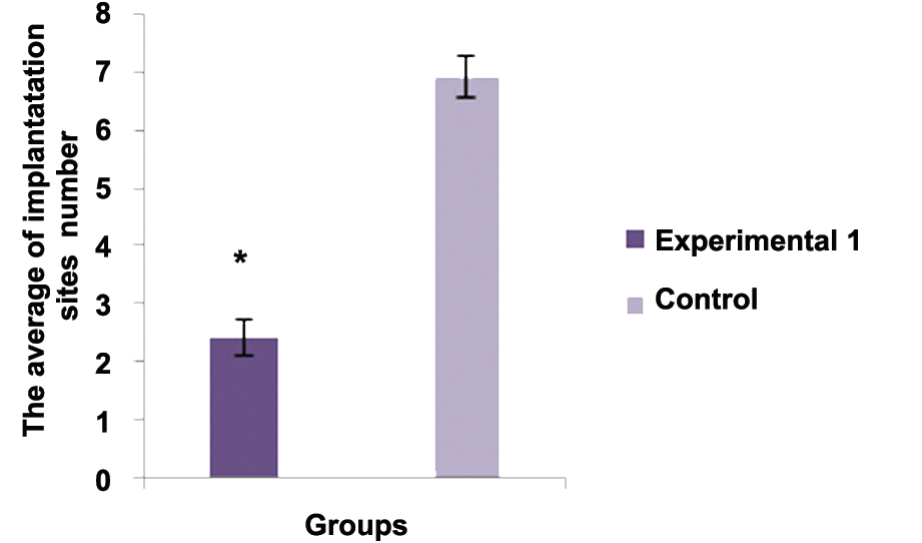
Comparison of the average number of implantation sites between experimental group 1 and control group. *P<0.001 as compared with control.

**Fig.2:**
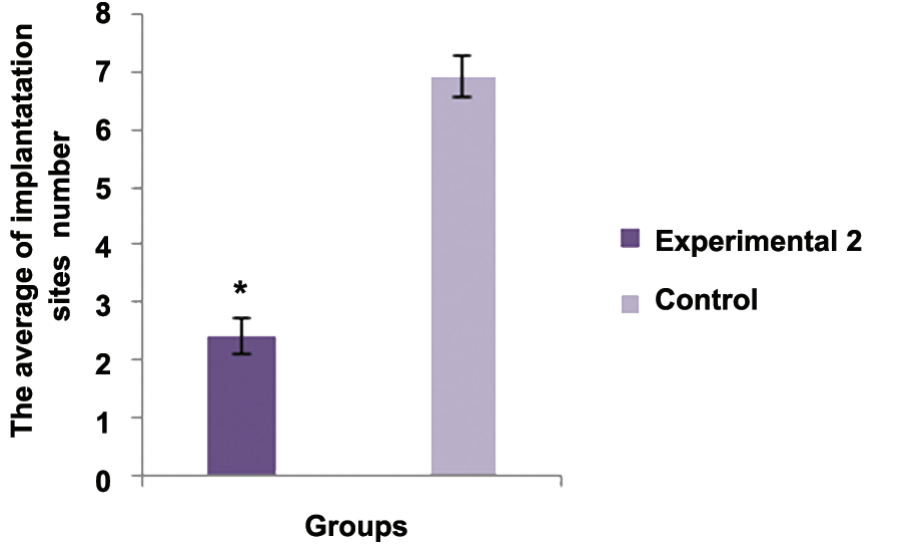
Comparison of average number of live births between experimental group 2 and control group. *P<0.001 as compared with control.

**Fig.3:**
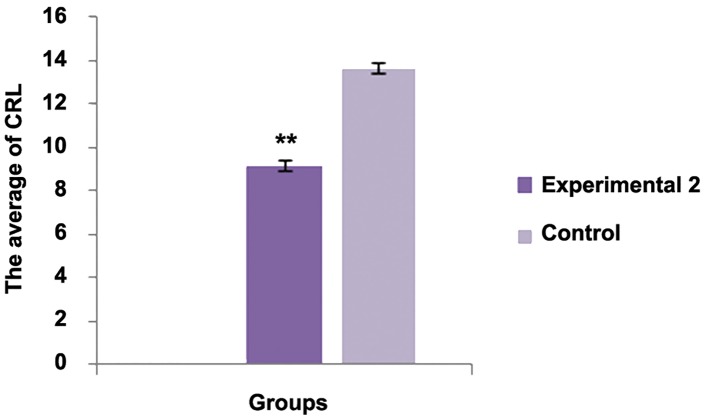
Comparison of the average birth weight between experimental group 2 and control group. There is no significant difference (P<0.05).

**Fig.4:**
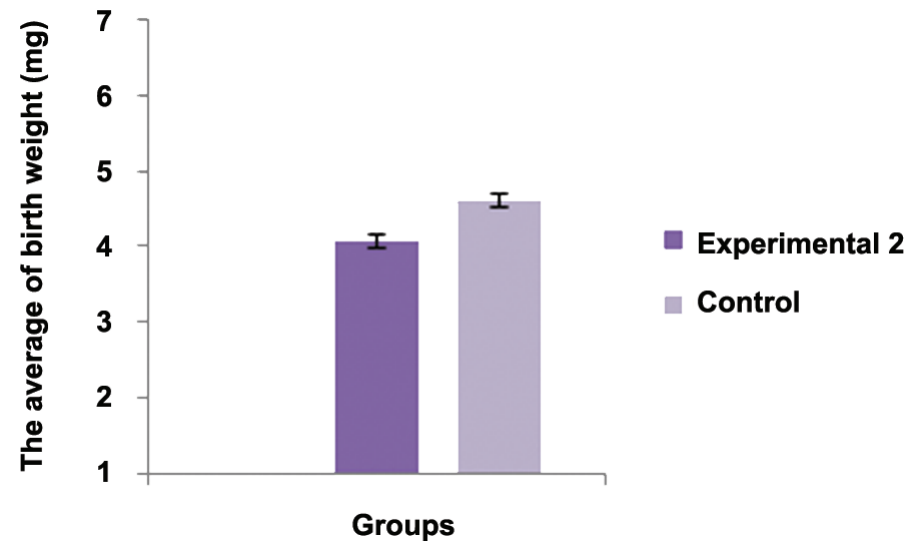
Comparison of the abnormality in neonates between experimental group 2 and control group. There is no significant difference (P<0.05).

**Fig.5:**
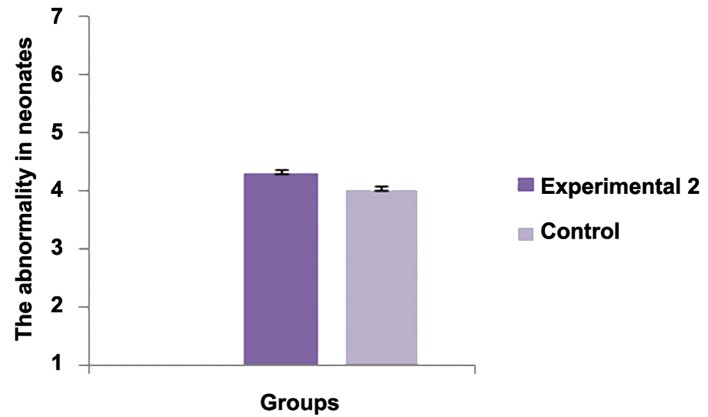
Comparison of the average of CRL between experimental group 2 and control group. **P<0.05 as compared with control.

The average number of the implantation sites between experimental group 1 and control group was statistically significant (P<0.001). The average number of live births between experimental group 2 and control group was statistically significant (P<0.001). There was no significant differences regarding the average birth weight between experimental group 2 and control group (P<0.05). There was no significant difference regarding abnormality between experimental group 2 and control group (P<0.05).There was significant difference regarding this parameter between experimental group 2 and control group [P<0.05, (Fig .1-5,Table1)]. 

CRL; Crown-rump length.

**Table 1:** Comparison of outcomes after administration of caffeine (150 mg/kg/day) between both experimental and control groups


Groups	Number	Number of implantation sites	Number of live births	Birth weight	Abnormality of neonates	CRL

Experimental 1	10	2.402 ± 0.3131*	-	-	-	-
Control	10	6.9 ± 0.3027	-	-	-	-
Experimental 2	10	-	2.2425 ± 0.1696*	4.0725 ± 0.076	4.32 ± 0.4509	9.41 ± 0.25**
Control	10	-	6.11 ± 0.3756	4.6075 ± 0.2649	4.4 ± 0.2160	13.67 ± 1.2252


**P<0.05 and *P<0.001 when compared to control values. Data are presented as mean ± SD. CRL; Crown-rump length.

## Discussion

In the present study, the administration of caffeine caused a significant decrease in implantation sites and number of live births. These results suggest that caffeine is likely to cause anti-fertility effect. The data also showed that caffeine consumption can decrease the birth weight of neonates that was not significant compared with the control group. Several fetuses were observed with a significant reduction in CRL.

Previous studies have shown that caffeine affects adversely human reproduction (14, 15). These effects could be very serious because most of the human populations of the world consumed caffeinecontaining foods (15). The findings of caffeine effects on pregnancy outcomes and fetal development differ widely; therefore, in the current study, developmental and reproductive risks of caffeine on pregnant rats and their offspring were evaluated.

In a recent study, Dorostghoal et al. (16) showed a reduction in ovarian weight and primordial follicle population caused by consumption of high dose caffeine during pregnancy and lactation, leading to diminish fertility and reproductive ability in rat. They stated that these alterations in the ovary were associated with a significant growth retardation of the female offspring.

Savineau and Mironneau (17) showed that absence of a caffeine-sensitive calcium-release channel in the sarcoplasmic reticulum prevents spasm of rat myometrium during pregnancy. Albina et al. (2) showed that caffeine administration to pregnant mice on gestational days 0-18 at doses of 120 mg/kg/day has adverse maternal effects that are evidenced by a significant reduction in body weight gain and gravid uterine weight. In consistent with their results, in the present study, caffeine decreased the birth weight, but it was not significant as compared with control group.

The results of the current work were in agreement with the results of Gilbert and Pistey (18). They showed that repeated IP injections of caffeine (4 to 16 mg/day) to pregnant rats resulted in significant resorptions and a decrease in the birth weight of neonates, but developmental malformations were not observed in neonates. In the current study, there was no significant difference regarding abnormality between experimental group 2 and control group, confirming previous investigations.

In the study by Gilbert and Rice (1), showed that the administration of different concentration of caffeine to monkeys before, during, and after pregnancy caused a dose-related increase in stillbirths and miscarriages as well as a decrease in maternal weight gain, suggesting that increased serum caffeine levels, particularly theophylline, may affect maternal physiology in pregnant monkey.

Vik et al. (19) found that a high caffeine intake in the third trimester of pregnancy was associated with an increased risk of small for gestational age (SGA) birth among male fetuses, but not in female fetuses. In current study, several fetuses were observed with small CRL and low birth weight that can be mediated by accumulation of caffeine in fetal tissues that could influence fetal development.

## Conclusion

IP injections of caffeine to pregnant rats resulted in a significant decrease in the numbers of implantation sites and live births. The findings showed that administration of caffeine induced anti-implantation activity, but the changes in abnormality and birth weight of the offspring were not significant. 
